# Effect of stimulated platelets in COVID-19 thrombosis: Role of alpha7 nicotinic acetylcholine receptor

**DOI:** 10.3389/fcvm.2022.1037369

**Published:** 2022-10-14

**Authors:** Lina Jankauskaite, Mantas Malinauskas, Ausra Snipaitiene

**Affiliations:** ^1^Institute of Physiology and Pharmacology, Lithuanian University of Health Sciences, Kaunas, Lithuania; ^2^Department of Pediatrics, Medical Faculty, Lithuanian University of Health Sciences, Kaunas, Lithuania

**Keywords:** COVID-19, SARS-CoV-2, platelets, inflammation, alpha7 nicotinic acetylcholine receptor (α7nAchR), thrombosis

## Abstract

Since early 2020, SARS-CoV-2-induced infection resulted in global pandemics with high morbidity, especially in the adult population. COVID-19 is a highly prothrombotic condition associated with subsequent multiorgan failure and lethal outcomes. The exact mechanism of the prothrombotic state is not well understood and might be multifactorial. Nevertheless, platelets are attributed to play a crucial role in COVID-19-associated thrombosis. To date, platelets' role was defined primarily in thrombosis and homeostasis. Currently, more focus has been set on their part in inflammation and immunity. Moreover, their ability to release various soluble factors under activation as well as internalize and degrade specific pathogens has been highly addressed in viral research. This review article will discuss platelet role in COVID-19-associated thrombosis and their role in the cholinergic anti-inflammatory pathway. Multiple studies confirmed that platelets display a hyperactivated phenotype in COVID-19 patients. Critically ill patients demonstrate increased platelet activation markers such as P-selectin, PF4, or serotonin. In addition, platelets contain acetylcholine and express α7 nicotinic acetylcholine receptors (α7nAchR). Thus, acetylcholine can be released under activation, and α7nAchR can be stimulated in an autocrine manner and support platelet function. α7 receptor is one of the most important mediators of the anti-inflammatory properties as it is associated with humoral and intrinsic immunity and was demonstrated to contribute to better outcomes in COVID-19 patients when under stimulation. Hematopoietic α7nAchR deficiency increases platelet activation and, in experimental studies, α7nAchR stimulation can diminish the pro-inflammatory state and modulate platelet reactiveness *via* increased levels of NO. NO has been described to inhibit platelet adhesion, activation, and aggregation. In addition, acetylcholine has been demonstrated to decrease platelet aggregation possibly by blocking the e p-38 pathway. SARS-CoV-2 proteins have been found to be similar to neurotoxins which can bind to nAChR and prevent the action of acetylcholine. Concluding, the platelet role in COVID-19 thrombotic events could be explained by their active function in the cholinergic anti-inflammatory pathway.

## Introduction

Since early 2020, severe acute respiratory syndrome coronavirus 2 (SARS-CoV-2)-induced infection resulted in global pandemics with high morbidity, especially in the adult population (1, 2). Coronavirus disease 19 (COVID-19) presented with a specific organ and system involvement, such as severe acute respiratory syndrome (SARS) which was already observed in other viral infections (3). In addition, this infection demonstrated very specific SARS-CoV-2-unique pathological phenotypes which raised a lot of concern and unanswered questions with regard to evidence-based management options (4, 5). Those clinical phenotypes do differ in patient clinical data on admission, complications, comorbidities, and clinical outcomes; thus, treatment might be tailored based on the clinical course and previous risk (5). Moreover, it emerged that COVID-19 is a highly prothrombotic condition associated with subsequent multiorgan failure and lethal outcomes (6–8). Multiorgan failure is still under investigation, yet different mechanisms such as endothelial cell damage, immune response, dysregulation of the renin-angiotensin-aldosterone system, and thromboinflammation have been involved (9, 10). A new type of COVID-19-associated multiorgan failure—a multisystem inflammatory syndrome (MISC) was described in children (11, 12). It closely resembles Kawasaki disease, known for several decades for its coronary complications (13). Up to 68% of affected children are treated in the pediatric intensive care unit (PICU) (14). Also, the increasing incidence of MISC is reported in young adults (15, 16). In most cases, MISC is characterized and investigated with the main focus on hyperinflammation, meanwhile, coagulation and thrombosis are less understood. Still, a study by Buonsenso et al. found D-dimers (fibrin degradation products) as an independent predictor of the outcomes of MISC (17). From the beginning of the COVID-19 pandemics, various data revealed that 20–50% of all COVID-19 hospitalized cases show abnormal coagulation results (18). An increase in D-dimer concentrations has been shown in a high percentage of severe COVID-19 cases. Elevated D-dimer values are associated with more severe diseases course and unfavorable outcomes of COVID-19 (8, 19–22). Platelets are another important marker in COVID-19. The most common finding in severe SARS-CoV-2-induced infection is thrombocytopenia. A meta-analysis by Jiang et al. demonstrated that lower platelet counts were detected in severe COVID-19 cases compared to milder ones (23). Thrombocytopenia has been reported to be associated with an increased risk of severe disease (24–26). Also, more studies analyse platelet activation role in the prothrombotic phenotype of COVID-19 patients. The exact mechanism of the prothrombotic state is not well understood and might be multifactorial. Nevertheless, platelets are attributed to play a crucial role in COVID-19-associated thrombosis. In this review, we will summarize the platelet role in COVID-19-associated thrombosis. Moreover, we will provide more insight into the role of the platelet alpha7 nicotinic acetylcholine receptor (α7nAChR) in the COVID-19-associated inflammation leading to thrombotic events.

## COVID-19 inflammation and thrombotic events: Clinical picture

The global pandemic of COVID-19 caused by SARS-CoV-2 started in 2020 and continues nowadays with the new disease entities. Initially, COVID-19 was thought to cause mainly respiratory symptoms which for the most affected were mild, subsequently, it had shown to be associated with a higher number of different complications.

Thrombosis plays a crucial part in the pathogenesis of COVID-19. In the beginning, SARS-CoV-2 infection induces a tremendous inflammatory reaction leading to uncontrolled or disrupted anti-inflammatory response (27). Interaction between SARS-CoV-2 and host cells, and prolonged inflammation cause endothelial damage and dysfunction with the result of excessive prothrombotic factor production contributing to an increased coagulation state. Moreover, COVID-19-induced hypoxia can further stimulate thrombosis through blood viscosity and hypoxia-inducible transcription factors (28). Nevertheless, DNA and histones from neutrophil extracellular traps (NETs) can additionally contribute to pro-thrombotic pathway activation (9, 29).

Up to 4.7% of severe COVID-19 cases progress to critically ill patients (30), and a significant number—approximately 79% result in severe thrombotic complications associated with a high mortality rate (8, 31, 32). Despite prophylactic anticoagulation treatment, almost one-third of the patients experience thrombotic events as demonstrated by the study of Lodigiani et al. (33). Moreover, a systematic review by Alahyari et al. revealed that thromboembolic events, such as deep vein thrombosis (DVT) or pulmonary embolism are most frequent of all the COVID-19-associated hematologic complications (34). Globally, a wide spectrum of incidence (10.9–58%) of DVT in COVID-19 patients was reported by several studies (33, 35, 36) with a higher percentage in critically ill patients (37). When compared to non-COVID acute respiratory distress syndrome (ARDS), COVID-19 ARDS demonstrated higher rates of pulmonary embolism (2.1 vs. 11.7%, respectively) (38, 39). A post-mortem study by Wichmann et al. revealed an important interplay between COVID-19 and venous thrombosis events (37). Most importantly, the unique feature of COVID-19-induced thrombosis is that it can be, both arterial and venous (32). Arterial thrombotic complications are less common (40), nevertheless, they can cause severe and devastating outcomes even with prescribed prophylactic anticoagulation therapy (41). A plethora of thrombotic complications are affecting cardiovascular and cerebrovascular systems (9, 10), myocardial infarction being the most prominent event (40, 42), and reaching 21% in the most recent meta-analysis study (43). Few studies suggested that ischemic stroke affects COVID-19 diseased younger people (6, 44, 45). Furthermore, the latest study by Xie et al. showed significantly higher cardiovascular outcomes after COVID-19 exposure (46), and cardiac complications have been linked to poor outcomes (43). Additionally, mesenteric ischemia is being reported in 1–5% of the cases with COVID-19 (38, 47). Besides macrovascular complications, more evidence demonstrates COVID-19-associated microvascular thrombotic events. Alveolar-capillary microthrombi have been found in severe COVID-19-induced ARDS cases (48–50). Nevertheless, more evidence shows that microangiopathy can cause complications in COVID-19 asymptomatic patients or patients with mild respiratory symptoms. An international study in perinatology recently demonstrated that pregnant women with mild COVID-19 symptoms resulted in placentitis leading to widespread placental insufficiency with subsequent fetal hypoxia and even lethal outcomes (51). In 37% of the examined placenta samples, multiple intervillous thrombi formations were identified and suggested as one of the contributing mechanisms to severe placental malperfusion. Another hypothesis of the possible presentation of SARS-CoV-2-induced microangiopathy was reported in several studies of case series showing increased incidence of “chilblains like” skin lesions during COVID-19 (52, 53). Moreover, this was supported by histological reports of skin biopsies where microthrombi were detected (54–56). The underlying hypothesis was SARS-CoV-2-associated epithelial damage, and secondary ischemia leading to the microangiopathic lesions (54). However, the clear confirmed pathogenesis and association of these skin lesions to COVID-19, especially in asymptomatic forms of the disease, is still under debate (57, 58). In general, the cause of various thrombosis in SARS-CoV-2 infection is closely related to coagulopathy, inflammation, platelet hyperactivity, thrombocytopathy, and endotheliopathy (9).

## Role of the platelets in the immune system, inflammation, and COVID-19-associated thrombosis

For a long time, platelets have been known as cells playing role in thrombosis and hemostasis. It is noteworthy that recently they have been attributed a significant role as immune mediators (59, 60). Platelets are a nucleate blood cells derived from megakaryocytes that reside primarily within the bone marrow (61). Additionally, studies have shown that the lung can be a potential site for platelet biogenesis. In the lung, platelets function as antiseptic cells when released in the vicinity of potential pathogen entry (62).

Patients with acute COVID-19 tend to be in a prothrombotic state and have severe inflammation (63). COVID-19 thrombosis encompasses both arterial and venous thromboembolic events, and they frequently co-occur with thrombocytopenia (32, 39). Systemic inflammation often leads to sepsis and septic shock and may present with increased platelet-leukocyte aggregates and thrombocytopenia (64–66). Genes encoding transcription factors involved in hematopoiesis and megakaryocyte biogenesis, such as Runt-related transcription factor 1 (RUNX1), GATA-binding factor 1 (GATA1), and others, have an impact on variations in platelet count (67–69). The liver produces thrombopoietin (TPO), which activates the TPO receptor in megakaryocytes to cause the creation of platelets through a process that is triggered by thrombocytopenia (70). In the final stage of platelet production, some of the transcription factors play a negative feedback role on TPO (71). Numerous cytokines can initiate megakaryopoiesis (e.g., interleukins 3, 6, and 11 (IL-3, IL-6, IL-11), fibroblast growth factor 4 (FGF4), and others) (72). Viruses can activate the host's cytokine profile to alter platelet formation through hepatic TPO synthesis. The simian immunodeficiency virus (SIV), which increases tumor growth factor (TGF), causes the synthesis of TPO (73); human papilloma virus-6 (HPV6) may prevent the development of TPO-induced megakaryocytic colonies (74). Meanwhile, SARS-CoV-2 *via* its spike protein may trigger the production of antibodies that cross-react with human TPO, to induce thrombocytopenia (75). Conversely, SAR2-CoV-2 stimulates angiotensin-converting enzyme (ACE) expression, which leads to induced inflammation *via* angiotensin II (Ang II) resulting in IL-6 stimulated TPO augmentation (76). In inflammation, IL-6 raises the levels of TPO to promote the creation of platelets (77), therefore, it can be considered that an inflammatory environment is required for COVID-19-induced thrombosis.

**Figure 1:**
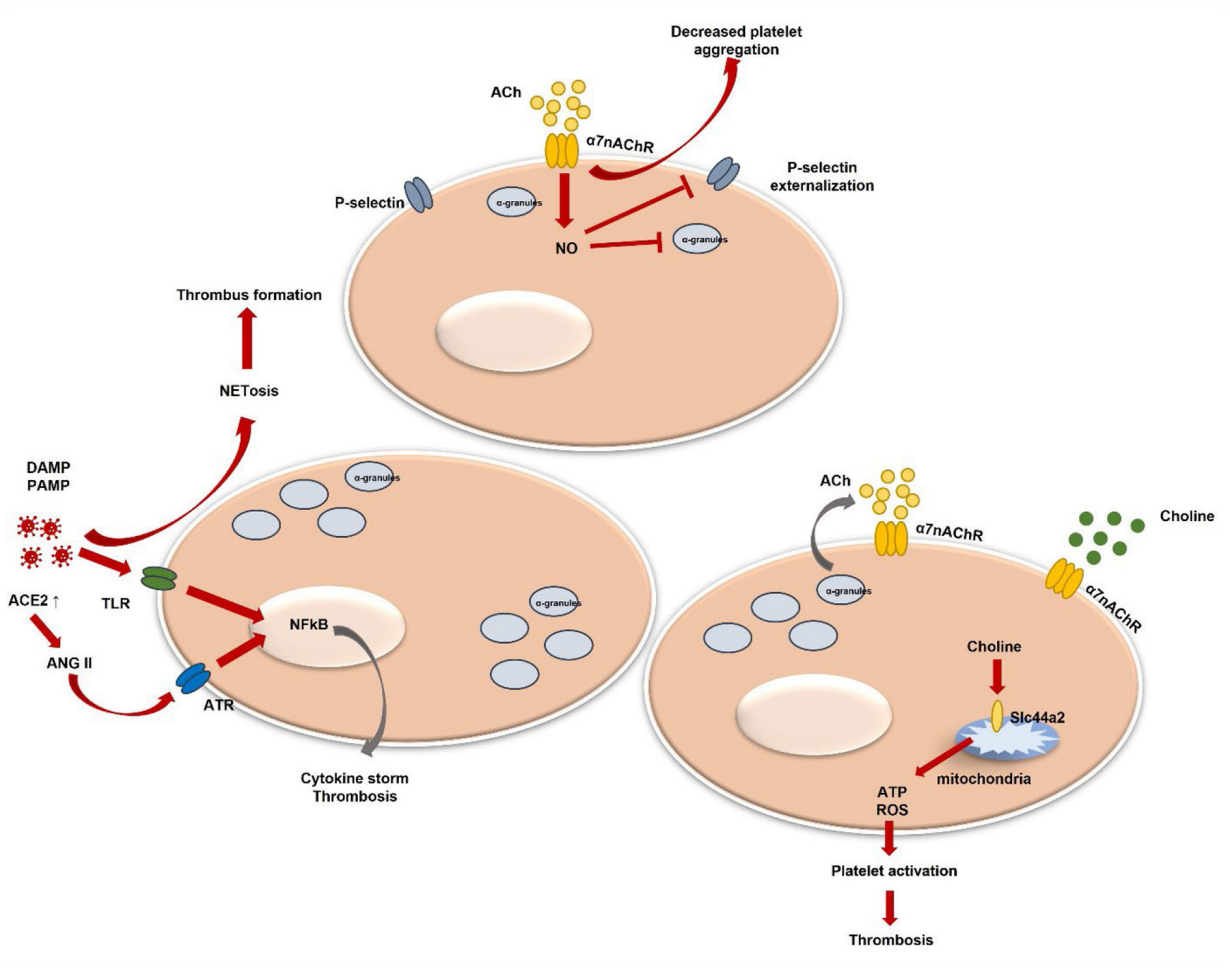
Platelet activation and α7nAChR involvement in thrombosis regulation. DAMPs, danger associated molecular patterns; PAMPs, pathogen associated molecular patterns; TLR, toll-like receptor; NF-kB, nuclear factor kappa-light-chain-enhancer of activated B cells; ACh, acetylcholine; NETosis, neutrophil extracellular trap formation; NO, nitric oxide; ACE2, angiotensin-converting enzyme-2; ANG II, angiotensin II; ATR, ANG II receptor; ATP, adenosine triphosphate; ROS, reactive oxygen species; α7nAChR, alpha7 nicotinic acetylcholine receptor.

Currently, more studies demonstrate direct viral-platelet interaction in platelet thrombotic and inflammatory function modulation (78, 79). Platelets do express pattern recognition receptors (PRRs), such as Toll-like receptors (TLR), Nod-like receptors, or C-type lectin receptors (80). Those receptors are crucial in damage-associated molecular patterns (DAMPs) and exogenous pathogen-associated molecular patterns (PAMPs) recognition. DAMPs and PAMPs are referred to as virus-associated molecular patterns (80–83). The attachment to DAMPs and PAMPs can initiate different intracellular pathways resulting in various pro-inflammatory cytokine production (84–87). In addition, platelet expression of functional TLR2 can further contribute to thrombotic pathway activation (84) (Figure 1). In the case of flu, the influenza virus has been proven to activate platelets *via* TLR-7 and Fcγ receptor IIa (FcγRIIa). The platelet expression of FcγRIIa leads to the activation of immune complexes (88). Antibodies against self-antigens, such as antiphospholipidic antibodies, have been reported in COVID-19 patients (89). However, thrombus formation was seen in COVID-19 patients' serum that had low levels of antiphospholipidic antibodies (90). Nevertheless, aberrant glycosylation of anti-SARS-CoV-2 spike immunoglobulin G(IgG) complexes was found to be a significant factor in the ability of these complexes to increase thrombus formation (91). Activation of TLR-7 evokes platelet degranulation, platelet-leucocyte aggregation, and NETosis stimulation leading to thrombus formation (79, 92) (Figure 1). Plasma from hospitalized COVID-19 patients demonstrated increased circulating platelet-neutrophil aggregates (93). Additionally, the autopsy of COVID-19 patients showed that microvascular thrombi composed of platelets and neutrophil extracellular traps were present (94). Also, recent studies show that platelets can internalize virus particles, and after viral ssRNA, dsRNA, or CpG DNA attachment to TLRs downstream signaling is initiated leading to platelet activation, platelet granule release, and P-selectin exposure (95, 96). P-selectin is a platelet receptor that has been linked to platelet activation. Platelets can bind to leukocytes *via* the P-selectin glycoprotein ligand-1 to mediate neutrophil rolling and intracellular leukocyte signaling (97, 98). Their depletion or blocking of the P-selectin-mediated interaction with neutrophils may reduce lung injury in COVID-19 (99). P-selectin, soluble CD40 ligand and others are released under platelet stimulation (100, 101). Their increased levels are observed in COVID-19 patients and P-selectin stimulates monocyte tissue factor (TF) expression contributing to a prothrombotic phenotype (102, 103).

Systemic levels of pro-inflammatory cytokines, such as TNFα, IL-1, and IL-6, are markedly elevated in severe COVID-19 (104). Moreover, the expression of pro-inflammatory cytokines, including TNFα, and IL-6 is dependent on Ang II (105, 106) (Figure 1), which amount is increased in SARS-CoV-2 infection (107). Angiotensin II (Ang II) contributes to endothelial dysfunction and the development of microvascular thrombosis (108), it stimulates TF expression, which is the physiological initiator of blood coagulation (109). Also, Ang II triggers platelet-derived growth factors (PDGF) production (110) and increases platelet aggregation (111). In addition, P-selectin expression levels are enhanced by activated platelets and by endothelial cells during Ang II stimulation (112). The relationship between Ang II and α7nAchR has been determined when activation of α7nAChR alleviated Ang II-mediated vascular smooth muscle senescence (113). Furthermore, it was suggested that decreased expression of α7nAchR might contribute to TNFα-induced vascular tissue inflammation, which was previously described as related to Ang II-mediated microvascular thrombosis (114). α7nAchR and the cholinergic system have been already studied regarding their beneficial role in COVID-19-induced hyperinflammation and disease outcomes (115, 116). Moreover, it has been shown that vagal stimulation *via* α7nAchR improves COVID-19-induced lung infection and inflammation, as well as systemic hyperinflammation (117–119). Additionally, patients lacking α7nAchR levels presented with higher C-reactive protein (CRP) values, more pronounced lymphopenia, extended pulmonary lesions, and increased expression of the TNFα pathway (115). Despite that α7nAchR role in platelets is still not widely studied and not well summarized, thus, we will analyse currently present data and platelet α7nAchR role in COVID-19-induced thrombosis.

## The cholinergic system, α7nAChR, and platelet role in COVID-19-induced hyperthrombosis

More and more data suggest that the autonomous nervous system plays a crucial role in inflammation *via* a cholinergic anti-inflammatory pathway (CAP). CAP is mainly composed of the parasympathetic nerves with the vagal nerve being most important together with acetylcholine (ACh) and its receptors (120). This pathway bridges the autonomic nervous system and immune system. Recently, the alleviating effect of COVID-19-induced hyperinflammation has been widely described in several studies (115, 117, 121–123). Under direct activation of the afferent vagal nerve, the main neurotransmitter ACh is released which further stimulates α7nAChR (124, 125) resulting in an anti-inflammatory response. Non-neuronal ACh was demonstrated to have an anti-inflammatory potential as well. α7nAChR is widely present on different immune cells and a variety of other cells, such as neuronal, endothelial cells, and platelets (126–128). Increased levels of TNFα, IL-6, and CRP have been detected in α7nAChR knockout mice (124, 129). Moreover, endothelial cell activation as well as leucocyte recruitment can be inhibited *via* α7nAChR stimulation (126). In addition, platelet function can be modulated during the inflammation process (128, 130).

Several studies have shown that platelets do contain some components of a non-neuronal cholinergic system, e.g., ACh, choline acetyltransferase, and acetylcholinestares (131–133). It is known that acetylcholinestares (AChE), for instance, hydrolyses the neurotransmitter ACh in the nervous system. Under AChE excess, an inflammatory process can be promoted (134). Three C-terminal variants of AChE have been identified (135). One of them—is a read-through transcript which is formed through the continuous transcription through intron I-4. This variant has been demonstrated to play an active role in the hematopoietic system and could be linked with its regulation under specific conditions, such as development or stress (132). Moreover, few studies found RNA signals of nAChR subunits, as well as α7 subunit in platelets (136, 137). Schedel et al. described functional α7nAChR Ca^2+^ channels in human platelets and in the megakaryocytic lineage and proposed an autocrine regulation mechanism *via* released stored ACh (128). Platelets are known to store various molecules in their granules. Those different cargo molecules are released under stimulation and contribute to coagulation, inflammation, or facilitating adhesion to other cells (138, 139). Nevertheless, ACh could be presented *via* other cells, such as endothelial cells, which are in close contact with platelets (131). A study by Bennett et al. indicated that endogenous ACh produced by platelets does inhibit platelet activation (140). It was demonstrated that *via* elevated nitric oxide (NO), ACh inhibits platelet degranulation, inhibits P-selectin externalization, and glycoprotein IIb IIIa (GPIIbIIIa) activation (141, 142) (Figure 1). Moreover, platelets express nitric oxide synthase 3 (NOS3) which may regulate platelets by an endogenous NO pathway (143). Few studies have shown that inhaled NO downregulates P-selectin, platelet aggregation, and fibrinogen binding in severe ARDS (144, 145). Kooijman et al. confirmed that mice lacking α7nAChR showed increased platelet aggregation *ex vivo* (130). Still, the hypothesis by authors has been raised that only a lack of both, platelet and endothelial α7nAChR could be associated with a significant impact on inflammation. Afterall, the role of endothelial cells in a cholinergic anti-inflammatory pathway is not well studied. Platelet and endothelial cell interaction is clearly described and shown to be important in various inflammatory conditions. Endothelial cell disruption during COVID-19 and released cytokines can be a possible mechanism of thrombosis (146). In addition, it has been demonstrated that platelets do play a crucial role in hypercoagulation during COVID-19 (147). Additionally, few studies revealed that monomeric C-reactive protein (mCRP) is linked with platelet activation which is mediated *via* p38 mitogen-activated protein kinase (MAPK) and Jun N-terminal kinase (JNK) (148). Interestingly, ACh has been shown to block mCRP binding and related pro-inflammatory action (149). MAPK is highly important in platelet activation, aggregation, and thrombus formation (150, 151). Moreover, the involvement and activation of p38 MAPK has already been widely described in SARS-CoV-2 (152). In addition, p38 MAPK can facilitate viral entry *via* ACE2 (153).

Interestingly, α7nAChR can be activated *via* choline as well (154). Choline is a precursor of ACh and phosphocholine (PC). It can efficiently act and is a relatively selective α7nAChR agonist (155). In COVID-19 patients, choline has been found to be downregulated, particularly in severe cases (156). Meanwhile, the intermediate product phosphatidylcholine has been detected to be upregulated. The possible underlying mechanism could be macrophage polarization associated with pathogen presentation (157). This further results in various cytokine secretion as a response to a COVID-19 infection. Another study showed that higher choline levels in pregnant women were associated with protective action against COVID-19 in fetal brain development (158). A recent study identified choline's role in platelet activation and thrombosis. The genetic loci including Slc44a2 have been already studied in thrombosis (159). Slc44a2 was demonstrated to mediate choline transport into mitochondria which results in mitochondrial oxygen consumption and ATP production (159). Mitochondrial dysfunction induces ATP decrease which results in decreased ATP release from platelets. Moreover, decreased ADP causes lower activation of platelets. Slc44a2 was already associated with venous thromboembolism (160, 161). In addition, Slc44a2 is well defined as a human neutrophil antigen (162). Moreover, it was proven to directly interact with platelet integrin α_IIb_β_3_ and trigger NETosis leading to thrombosis (163, 164) (Figure 1). PC is nAChR agonist as well (165). Studies showed its inhibitory potential for IL-1beta release from monocytes in α7nAChR dependent manner (166). Furthermore, PC epitopes are exposed on various pathogens and their interaction with host proteins, such as platelet-activating factor receptors (167, 168) leads to pathogen adhesion to the surface of the host cell and cell invasion (169, 170). Nevertheless, less is known regarding PC function and excretion from platelets and involvement in SARS-CoV-2 or other viral pathogen-induced inflammation and/or thrombosis. To date, only one study defined that platelets could release choline metabolites under stimulation (171).

## Conclusion

Concluding, the prothrombotic state of COVID-19 is multifactorial, nevertheless, platelets do play an important role in inducing COVID-19 hypercoagulation and thrombosis. Due to their different secretory factors which induce coagulation and inflammation, they participate in thrombosis induction *via* different pathways. One of the possible and less studied is the cholinergic system and platelet α7nAChR which has been less studied but could be a very significant part in SARS-CoV-2-induced infection. As previously shown, nervus vagus stimulation can benefit COVID-19-associated hyperinflammation, thus, *via* platelet α7nAChR it might decrease coagulation and thrombotic process together with decreased inflammatory factors (which additionally activate platelets) and benefit COVID-19 patients. Different studies show that platelets can produce choline products under stimulation, thus, α7nAChR could be stimulated leading to its beneficial anti-inflammatory and possible anti-thrombotic effect. However, more studies are necessary to confirm this hypothesis.

## Author contributions

LJ: hypothesis, editing, visualization, and supervision. LJ, MM, and AS: analysis, writing original draft, and review. All authors contributed to the article and approved the submitted version.

## Funding

Publishing costs were partially covered by a local fund of Scientific center of Lithuanian University of Health Sciences.

## Conflict of interest

The authors declare that the research was conducted in the absence of any commercial or financial relationships that could be construed as a potential conflict of interest.

## Publisher's note

All claims expressed in this article are solely those of the authors and do not necessarily represent those of their affiliated organizations, or those of the publisher, the editors and the reviewers. Any product that may be evaluated in this article, or claim that may be made by its manufacturer, is not guaranteed or endorsed by the publisher.
